# A 6-yr evaluation of prescribed-fire timing on yearling cattle growth performance and plant community dynamics on native tallgrass prairie in the Kansas Flint Hills

**DOI:** 10.1093/tas/txad129

**Published:** 2023-11-18

**Authors:** Z M Duncan, A J Tajchman, J Lemmon, W R Hollenbeck, D A Blasi, W H Fick, K C Olson

**Affiliations:** Department of Animal Sciences and Industry, Kansas State University, Manhattan, KS 66506, USA; Department of Animal Sciences and Industry, Kansas State University, Manhattan, KS 66506, USA; Department of Animal Sciences and Industry, Kansas State University, Manhattan, KS 66506, USA; Department of Animal Sciences and Industry, Kansas State University, Manhattan, KS 66506, USA; Department of Animal Sciences and Industry, Kansas State University, Manhattan, KS 66506, USA; Department of Agronomy, Kansas State University, Manhattan, KS 66506, USA; Department of Animal Sciences and Industry, Kansas State University, Manhattan, KS 66506, USA

**Keywords:** grazing, *Lespedeza cuneata*, prescribed fire, rangeland plant composition, stocker cattle growth performance

## Abstract

A 6-yr experiment was conducted to determine the effects of prescribed-fire season on stocker cattle growth performance and rangeland plant community characteristics in the Kansas Flint Hills. Eighteen pastures were grouped by watershed and each watershed was randomly assigned to 1 of 3 prescribed-fire treatments: spring (11 April ± 5.7 d), summer (25 August ± 6.2 d), or autumn (2 October ± 9.0 d). All burns were applied prior to grazing in years 1, 2, 3, and 5; however, no burns were applied in year 4 because of unfavorable burn conditions. Over 5 consecutive grazing seasons, 1,939 yearling stocker calves (initial BW = 281 ± 58.9 kg) were grazed from May to August at a targeted stocking density of 280 kg live-weight + ha^−1^. Beginning in June of 2018 (pretreatment), a permanent 100-m transect was established in each pasture and was used to determine plant-species composition using a modified step-point method. Forage biomass accumulation and root carbohydrate concentrations of 4 native tallgrass plant species were also measured. All data were analyzed as a completely randomized design using a mixed model. Average daily gain (ADG) was 0.05 to 0.07 kg greater (*P* = 0.02) for calves grazing spring-burned pastures compared with calves grazing summer- or autumn-burned pastures; however, ADG did not differ (*P* ≥ 0.55) between calves assigned to the summer or autumn prescribed-fire treatments. Basal cover of all graminoids and all forbs did not differ (*P* ≥ 0.30) among prescribed-fire treatments; however, basal cover of C3 grasses tended (*P* = 0.06) to be greater while basal cover of C4 grasses tended (*P* = 0.08) to be less in autumn-burned pastures compared with spring-burned pastures. Forage biomass accumulation did not differ (*P* = 0.58) among treatments. In addition, root starch or root water-soluble carbohydrate concentrations in big bluestem (*Andropogon gerardii*), little bluestem (*Schizachyrium scoparium*), Indiangrass (*Sorghastrum nutans*), or purple prairieclover (*Dalea purpurea*) did not differ (*P* ≥ 0.26) among prescribed-fire treatments. Overall, we interpreted these data to suggest that prescribed-fire timing had small influences on yearling stocker cattle growth performance and rangeland plant composition but did not influence forage biomass accumulation or root carbohydrate concentrations of key native tallgrass plant species in the Kansas Flint Hills.

## Teaser

A 6-yr experiment was conducted to evaluate the effects of unconventional applications of prescribed fire on growth performance of grazing yearling beef cattle and plant community composition in native tallgrass prairie. We present evidence that summer-season prescribed fire allowed similar final body weights for grazing yearling cattle and produced positive to neutral effects on native plant populations compared to conventional spring-season prescribed fire.

## Introduction

The Kansas Flint Hills are the largest intact remnant of tallgrass prairie in the world (Samson and Knopf, 1994). During Euro-American settlement, the shallow, rocky soils made the area unsuitable for cultivation; however, ranchers in the mid-1800s realized that cattle grazing native warm-season grasses in the region could achieve weight gains of 90 to 136 kg during the growing season. In addition, cattle that grazed burned pastures had greater weight gains compared with those that grazed non-burned pastures (Anderson, 1953). Early research evaluating prescribed-fire timing led to the almost-exclusive recommendation that fire should be applied in mid- to late April because of improvements in stocker cattle weight gains (Smith and Owensby, 1978), increased native warm-season grass production (Anderson et al., 1970; Towne and Owensby, 1984), and reductions in invasive woody-stemmed plant species (Owensby et al., 1973).

Today, ~850,000 ha of native Flint Hills rangeland are burned annually between mid-March and early May (KDHE, 2022). Fires applied in March and April accounted for 74% to 93% of annual prescribed-fire detections in the Flint Hills between 2007 and 2018 (Baker et al., 2019). Although spring-season prescribed fire is widely practiced in the Flint Hills, smoke management during this period can be difficult. Strong spring-season winds and low relative humidity typically reduce the number of days available to safely conduct a burn to the extent that up to 40,000 ha can be burned in a single day (Baldwin et al., 2022). Smoke produced from burning large amounts of rangeland can travel to urban areas downwind of the Flint Hills and reduce air quality. Smoke contains fine particulate matter and precursors for ozone which can negatively impact human health (KDHE, 2010).

Another challenge associated with spring-season prescribed fire is the inability of spring fire to control sericea lespedeza (*Lespedeza cuneata*). Sericea lespedeza is a perennial legume that was originally brought to southeast Kansas in the 1930s (Ohlenbusch et al., 2007). Soon after its introduction, sericea lespedeza expanded into Kansas and has degraded ~190,000 ha of native rangeland, most of which is located in the Flint Hills (KDA, 2022). The Flint Hills are predominantly grazed by beef cattle and attempts to manage sericea lespedeza infestations via grazing have been ineffective because high concentrations of condensed tannins in the plant discourage herbivory by cattle (Preedy et al., 2013; Sowers et al., 2019). In addition, spring-season prescribed fire may stimulate sericea lespedeza germination which further promotes its establishment in native rangelands (Wong et al., 2012).

Currently, Flint Hills ranchers typically apply a spring-season prescribed fire in April and then graze yearling beef cattle at a high relative stocking density (i.e., intensive-early stocking) for 75 to 100 d. Intensively grazing mature ewes for 60 d after cattle grazing reduced basal cover of sericea lespedeza, reduced sericea seed production, and appeared to be a sustainable strategy for sericea control (Lemmon et al., 2023); however, this practice has not been widely adopted in the Flint Hills. Herbicide application temporally reduced basal cover of sericea lespedeza; however, routine use of herbicides can have negative impacts on non-target native forbs (Gatson, 2018).

When prescribed fire application was shifted from April to August or September, sericea lespedeza basal cover, biomass, and seed production were sharply reduced compared with traditional spring-season prescribed fire. In addition, native forb diversity was greater in plots burned in August or September compared with those burned in April (Alexander et al., 2021). Similar experiments have also reported improved forb diversity, reduced cover of woody plants (Weir and Scasta, 2017) and reduced cover of yellow bluestem (*Bothriochloa ischaemum*; Reemts et al. 2019) when fire was applied in late summer or early autumn (i.e., September-October). Widespread adoption of growing season prescribed fires in the Kansas Flint Hills have met resistance because of perceived effects of fire applied later in the year on stocker cattle growth performance and possible protracted stress on warm-season forage grasses. Therefore, the objectives of this experiment were to document the effects of prescribed fires applied in spring, summer, or autumn on stocker cattle growth performance, rangeland plant composition, forage biomass accumulation, and root carbohydrate concentrations in key native tallgrass plant species over a 6-yr period.

## Materials and Methods

The Kansas State University Institutional Animal Care and Use Committee reviewed and approved all animal handling and animal care practices used. All animal procedures were conducted in accordance with the Guide for the Care and Use of Animals in Agricultural Research and Teaching (FASS, 2010).

Our experiment was conducted at the Kansas State University Beef Stocker Unit between June 2018 and August 2023. Eighteen pastures were grouped by watershed and each watershed was assigned randomly to 1 of 3 prescribed-fire treatments: spring (11 April ± 5.7 d), summer (25 August ± 6.2 d), or autumn (2 October ± 9.0 d). Pastures ranged in size from 16 to 30 ha and had previously been managed using annual spring-season prescribed fire followed by a 90-d intensive-early grazing season. Burn treatments were applied prior to grazing in years 1, 2, 3, and 5; however, no burn treatments were applied in year 4 due to unfavorable burn conditions. A more detailed description of the study location and burn conditions used to conduct the experiment is described by Duncan et al. (2021).

### Animal performance.

A total of 1,939 yearling beef calves (initial BW = 281 ± 58.9 kg) were grazed over 5 consecutive growing seasons. Calves were grazed for 90 d from May to August at a targeted stocking density of 280 kg live weight ha^−1^. Based on cattle availability, heifers were grazed in year 1 and steers were grazed in years 2 to 5. Initiation and termination of grazing varied slightly from year to year based on cattle availability. At receiving, calves were individually weighed using a hydraulic squeeze chute (Silencer, Moly Manufacturing Inc., Lorraine, KS). Initial body weights (BW) were recorded, visual identification tags were applied, and calves were stratified by weight and randomly assigned to pasture and treatment. All cattle were fed a high-roughage growing diet at 2.0% of BW for a minimum of 14 d until turnout for grazing. The day grazing began, calves were reweighed individually to determine initial BW. In addition, all calves received a growth-promoting implant (Ralgro, Merck Animal Health, Rahway, NJ). At the completion of the grazing season, each pasture was gathered and individual weights were immediately measured. The livestock scale used to measure individual weights was validated annually in April (Salina Scale, Inc. Salina, KS).

### Botanical composition.

In 2018 (pretreatment), a permanent 100-m transect was established in each pasture. Transects were established exclusively on Benfield-Florence complex soils in areas with less than 2% slope. Each transect point (i.e., endpoints and center) was marked with orange survey stakes (Forestry Suppliers, Inc., Jackson, MS) and GPS coordinates were recorded (Garmin eTrex 20x, Olathe, KS). Soil cover and botanical composition was evaluated annually in June along each transect using a modified step-point technique (Owensby, 1973; Farney et al., 2017). Using a step-point device, a point was randomly selected on the ground at 2-m intervals along both sides of each transect (i.e., 100 total points per transect; Owensby, 1973). Each point was first characterized as a hit on bare soil, litter, or live basal plant matter to determine soil cover. Next, the closest rooted plant (i.e., grass, forb, or shrub) in a 180° arc in front of the selected point was recorded. If the closest rooted plant was a grass, then the closest rooted forb or shrub was recorded. Plant species composition was then calculated as described by Farney et al. (2017). Common and scientific names were those recommended by Haddock (2005).

Plant species were grouped into categories based on their growth form as described by Hickman et al. (2004). Categories included total C4 grasses, C4 perennial tall grasses, C4 perennial mid-grasses, C4 perennial short grasses, C3 perennial grasses and sedges, annual forbs, perennial forbs, and shrubs. In addition, plant species were also categorized as native graminoids, introduced graminoids, native forbs, introduced forbs, leguminous forbs, nectar-producing forbs, increaser shrubs (i.e., shrubs that tend to proliferate in response to grazing; Vesk and Westoby, 2001), and nectar-producing shrubs.

### Forage biomass.

Forage biomass accumulation was evaluated in late June to early July in 2018, 2020, and 2022. Ten 50 × 50-cm clipping frames were randomly placed alongside each transect at 10-m intervals beginning at the south or east end. Once placed, litter from the previous growing season was removed and all remaining vegetation was clipped 1 cm above the soil surface. All samples were weighed, dried in a forced-air oven (50 °C; 96 h), and reweighed to estimate standing forage dry matter ha^−1^.

### Root carbohydrate reserves.

Root starch and root water-soluble carbohydrate concentrations of big bluestem (*Andropogon gerardii*), little bluestem (*Schizachyrium scoparium*), Indiangrass (*Sorghastrum nutans*), and purple prairieclover (*Dalea purpurea*) were measured in mid-June from 2018 to 2021. A steel spading fork (Bully Tools, Steubenville, OH) was used to collect ~60 g of roots and rhizomes to a depth of 20 cm from each targeted species within each pasture. Following collection, samples were placed in bags within individual plant species and stored in coolers. Subsequently, samples were washed with tap water, separated from the aerial portion of the plant, and dried in a forced-air oven (50 °C; 96 h). Once dry, samples were sent to a commercial laboratory (Dairy 1, Ithaca, NY) for root starch and water-soluble carbohydrate analysis. Water-soluble carbohydrate concentrations were determined as described by Hall et al. (1999) using a Thermo Scientific Genesys 10s Vis Spectrophotometer. Root starch concentrations were determined by incubating samples in a water bath at 40 °C and filtering them through Whatman no. 41 filter paper. Residues were then autoclaved, incubated with a glucoamyalse enzyme, and analyzed using a YSI 2700 SELECT Biochemistry Analyzer (YSI Inc. Life Sciences, Yellow Springs, OH).

### Statistical analysis.

All data were analyzed as a completely randomized design using a mixed-model (PROC MIXED; SAS 9.4, SAS Inst. Inc., Cary, NC). Class variables included burn treatment, year, and pasture. The initial model contained fixed effects for treatment, year, and treatment × year and a random effect for pasture within treatment. No treatment × year interactions were significant (*P* > 0.10) for yearling growth performance, forage biomass accumulation, root carbohydrate concentrations, or major classifications of range plants; therefore, the final models relevant to those data categories contained a term for treatment only as a fixed effect and year and pasture within treatment as random effects. In the case of individual range plant species, occasional treatment differences or tendencies (treatment × time—*P* ≤ 0.10) in basal cover were observed over time. As these phenomena were not associated with temporal trends in basal cover and appeared to be ephemeral, main-effect means of basal cover were reported for hairy grama (*Bouteloua hirsuta*), Indiangrass, Kentucky bluegrass (*Poa pratensis*), prairie junegrass (*Koeleria macrantha*), sideoats grama (*Bouteloua curtipendula*), leadplant (*Amorpha canescens*), and New Jersey tea (*Ceanothus americanus*).

When protected by a significant *F*-test (*P* ≤ 0.05), treatment means were separated using the method of least significant difference. Significance was declared at *P* ≤ 0.05 and tendencies at 0.05 ≤ *P* ≤ 0.10.

## Results and Discussion

### Animal performance.

After 5 consecutive grazing seasons total BW gains and ADG were greater (*P* = 0.02; Table 1) for calves grazing spring-burned pastures compared with calves grazing summer- or autumn-burned pastures; however, total BW gains and ADG were similar (*P* ≥ 0.55) among calves grazing summer- and autumn-burned pastures. Total BW gains during the 90-d grazing period averaged 96.6, 91.5, and 90.3 kg for the spring, summer, and autumn prescribed-fire treatments, respectively. Initial BW did not differ (*P* = 0.23) among treatments; moreover, final BW did not differ (*P* = 0.36) between spring and summer prescribed-fire treatments but both were greater (*P* = 0.02) than that of the autumn prescribed-fire treatment.

**Table 1.** Effects of prescribed-fire season on yearling stocker cattle growth performance and forage biomass accumulation in the Kansas Flint Hills

	Prescribed fire season^1^				
Item	Spring	Summer	Autumn	SEM^2^	*P*-value^3^
Initial bodyweight, kg	287	290	285	2.7	0.23
Final bodyweight, kg	384^a^	382^a^	376^b^	2.5	0.01
Total bodyweight gain^4^, kg	96.6^a^	91.5^b^	90.3^b^	2.10	0.02
Average daily gain^5^, kg d^−−1^	1.07^a^	1.02^b^	1.00^b^	0.023	0.02
Forage biomass^6^, kg ha^−1^	1,968	2,151	2,210	240.4	0.58

^1^Eighteen pastures were grouped by watershed and randomly assigned to 1 of 3 prescribed-fire treatments: spring (11 April ± 5.7 d), summer (25 August ± 6.2 d), or autumn (2 October ± 9.0 d). Yearling beef cattle were grazed on all pastures from May to August at a targeted stocking density of 280 kg live-weight ha^−1^ following prescribed fire application.

^2^Mixed-model standard error of the mean (SEM) associated with comparison of treatment main-effect means.

^3^Treatment main effect.

^4^Calculated as final body weight—initial body weight.

^5^Calculated as total body weight gain ÷ total grazing days.

^6^Measured in late June to early July in 2018, 2020, and 2022.

^a,b^Within rows, means with unlike superscripts differ (*P* ≤ 0.05).

Previous reports indicated that yearling stocker cattle weight gains were greater for cattle grazing burned pastures compared with non-burned pastures (Woolfolk et al., 1975; Svejcar, 1989; McCollum et al., 1992). Early research in the Kansas Flint Hills suggested that ranchers should apply prescribed fire during mid- to late April to maximize growth performance under intensive-early stocking management (Anderson et al., 1970; Smith and Owensby, 1978). In our experiment, total BW gains were 5.1 to 6.3 kg less for calves grazing summer- and autumn-burned pastures compared with calves grazing spring-burned pastures, respectively. McMillan et al. (2022) observed a 24-kg reduction in BW gain in yearling cattle grazing pastures burned in August–September compared with pastures burned in March–April. In that experiment, calves were grazed from April to September and burn treatments were applied to one-third of the pasture each year; growing-season burns were not applied prior to grazing in year 1 but were subsequently applied during the grazing season throughout the remainder of the three-year experiment. In our experiment, grazing was always preceded by fire application. Differences in study design likely contributed to the difference in weight gains observed for stocker cattle grazing pastures burned during the growing season.

### Soil cover.

Differences in cattle growth performance among prescribed-fire treatments may have been associated with diet quality. Proportions of litter on the soil surface were greatest (*P* ≤ 0.04; Table 2) in the summer prescribed-fire treatment, intermediate (*P* ≤ 0.04) in the autumn prescribed-fire treatment, and least (*P* ≤ 0.01) in the spring prescribed-fire treatment. Overall, litter on the soil surface was 6.5% and 11.6% greater in ­autumn- and summer-burned pastures, respectively, compared with spring-burned pastures. Diets selected by calves assigned to the autumn or summer prescribed-fire treatments may have contained small amounts of dead plant material from the previous growing season which could have contributed to the slightly reduced growth performance we observed.

**Table 2.** Effects of prescribed-fire season on proportions of bare soil, litter on the soil surface, and basal plant cover on native tallgrass prairie measured annually in June

	Prescribed fire season^1^				
Item, % total area	Spring	Summer	Autumn	SEM^2^	*P*-value^3^
Bare soil	66.0^a^	55.7^b^	59.8^b^	2.40	<0.01
Litter cover	20.7^c^	32.3^a^	27.2^b^	2.33	<0.01
Basal vegetation cover	13.3	12.0	13.0	0.70	0.19

^1^Eighteen pastures were grouped by watershed and randomly assigned to 1 of 3 prescribed-fire treatments: spring (11 April ± 5.7 d), summer (25 August ± 6.2 d), or autumn (2 October ± 9.0 d). Yearling beef cattle were grazed on all pastures from May to August at a targeted stocking density of 280 kg live-weight ha^−1^ following prescribed fire application.

^2^Mixed-model standard error of the mean (SEM) associated with comparison of treatment main-effect means.

^3^Treatment main effect.

^a,-c^Within rows, means with unlike superscripts differ (*P* ≤ 0.05).

Conversely, proportions of bare soil were greater (*P* ≤ 0.01; Table 2) in the spring prescribed-fire treatment compared with the summer or autumn prescribed-fire treatments. Soil cover was measured annually in June; therefore, as the length of time between fire application and sample collection increased, proportions of litter on the soil surface increased while proportions of bare soil decreased. Despite this observation, basal vegetation cover did not differ (*P* = 0.19) among pastures burned in spring, summer, or autumn and accounted for 12% to 13.3% of total area.

### Botanical composition.

Basal cover of total graminoids represented 86% to 89% of total basal plant cover and did not differ (*P* = 0.30; Table 3) among prescribed-fire treatments. Similarly, basal cover of native and introduced graminoids did not differ (*P *≥ 0.24) among spring-, summer-, or autumn-burned pastures; however, prescribed-fire season tended to influence relative basal cover of C3 and C4 grasses. Basal cover of C3 grasses tended to be greater (*P *= 0.06) in the autumn prescribed-fire treatment, intermediate in the summer prescribed-fire treatment, and least in the spring prescribed-fire treatment.

**Table 3.** Effects of prescribed-fire season on graminoid composition in native tallgrass prairie measured annually in June

	Prescribed fire season^1^				
Item, % basal plant cover	Spring	Summer	Autumn	SEM^2^	*P*-value^3^
Total graminoid cover	89.4	89.3	85.8	2.53	0.30
Native graminoids	85.0	85.2	80.0	3.41	0.24
Introduced graminoids	4.3	4.1	5.8	2.46	0.74
C3 grasses	19.2^z^	26.1^yz^	27.2^y^	3.44	0.06
Sedges	13.9	19.6	16.3	2.80	0.15
Kentucky bluegrass	2.0^b^	4.1^ab^	5.8^a^	1.25	0.02
Prairie junegrass	0.9^z^	1.3^yz^	2.5^y^	0.69	0.08
C4 grasses	70.1^y^	63.2^yz^	58.7^z^	4.79	0.08
Tallgrasses	33.8	37.3	35.1	2.37	0.35
Big bluestem	20.5	19.9	22.1	1.59	0.62
Indiangrass	12.5^b^	15.9^a^	11.5^b^	1.19	<0.01
Mid-grasses	33.1^a^	25.0^b^	22.3^b^	3.73	0.02
Little bluestem	14.5^a^	7.9^b^	10.7^ab^	2.15	0.03
Sideoats grama	13.0^ab^	14.9^a^	7.8^b^	2.68	0.04
Yellow bluestem	2.2	0.0	0.0	2.06	0.45
Shortgrasses	3.1^a^	0.8^b^	1.2^b^	0.83	0.03
Hairy grama	2.3^y^	0.6^yz^	0.5^z^	0.79	0.08

^1^Eighteen pastures were grouped by watershed and randomly assigned to 1 of 3 prescribed-fire treatments: spring (11 April ± 5.7 d), summer (25 August ± 6.2 d), or autumn (2 October ± 9.0 d). Yearling beef cattle were grazed on all pastures from May to August at a targeted stocking density of 280 kg live-weight ha^−1^ following prescribed fire application.

^2^Mixed-model standard error of the mean (SEM) associated with comparison of treatment main-effect means.

^3^Treatment main effect.

^a,b^Within rows, means with unlike superscripts differ (*P* ≤ 0.05).

^y,z^Within rows, means with unlike superscripts tended to differ (*P* ≤ 0.10).

The trend toward increased basal cover of C3 grasses in autumn-burned pastures was associated with temporal changes to Kentucky bluegrass (non-native) and prairie junegrass (native) populations. Kentucky bluegrass basal cover was greater (*P* < 0.02; Table 3) in pastures burned in autumn compared with pastures burned in spring; summer-burned pastures were intermediate to and not different from those burned in spring or autumn. A similar trend was observed for basal cover of prairie junegrass where it tended (*P* = 0.08) to be greater in autumn-burned pastures compared with spring-burned pastures.

Towne and Owensby (1984) and Towne and Kemp (2008) reported a reduction in basal cover of Kentucky bluegrass following fire application in March, April, July, or December. Conversely, basal cover of prairie junegrass and sedges increased when fire was applied in February or November (Towne and Craine, 2014). In addition, basal cover of sedges was 19.7% greater in watersheds burned in late July or early August every other year compared with watersheds burned annually in April (Towne and Kemp, 2008). Overall, it appears that shifting prescribed fire from April to August or October resulted in minor changes to basal cover of certain C3 graminoid species. Sedge species, the predominant C3 graminoids in the region (Table 3), accounted for 1.9% to 3.4% of grazed yearling-steer diets in the Kansas Flint Hills (Sowers et al., 2019), indicating that stocker cattle will consume C3 forages, including sedges, if they are available. In addition, increased presence of C3 grasses could potentially extend the current Flint Hills grazing season outside of the traditional May to August period.

Total basal cover of C4 grasses tended (*P* = 0.08; Table 3) to be greatest in spring-burned pastures, intermediate in summer-burned pastures, and least autumn-burned pastures. Within C4-grass growth forms, basal cover of total C4 tallgrasses did not differ (*P *= 0.35) among prescribed-fire treatments; however, prescribed-fire season appeared to influence basal cover of Indiangrass. Basal cover of Indiangrass was greater (*P* < 0.01) in the summer prescribed-fire treatment compared with the spring or autumn prescribed-fire treatments. Alexander et al. (2021) observed a similar trend where basal cover of Indiangrass was greatest in plots burned in August, intermediate in plots burned in April, and least in plots burned in September. Indiangrass produces biannual tillers and the percentage of first-year tillers are greatest toward the end of the growing season (McKendrick et al., 1975). Fire applied in September or October could potentially damage first-year tillers and reduce the propagation potential of Indiangrass populations.

Basal cover of C4 mid- and shortgrasses was greater (*P* ≤ 0.03; Table 3) in spring-burned pastures compared with summer- or autumn-burned pastures. Within C4 mid-grasses, basal cover of little bluestem was less (*P* < 0.01) in the summer prescribed fire treatment compared with the spring prescribed-fire treatment. Conversely, basal cover of sideoats grama was greatest (*P* = 0.04) in summer-burned pastures, least in autumn-burned pastures, and intermediate in spring-burned pastures. Reemts et al. (2019) reported that cover of yellow bluestem, a non-native C4 mid-grass, was reduced in plots treated with late-summer prescribed fire compared with non-burned plots. Basal cover yellow of bluestem did not differ (*P* = 0.45) among prescribed-fire regimes evaluated in our experiment likely due to small relative cover values; however, it was present within all pastures at the initiation of our prescribed-fire treatments in 2018 (data not shown). After repeated prescribed-fire applications, yellow bluestem remained along transects treated with spring fire but was not detected along transects treated with summer or autumn fire. Within C4 shortgrasses, basal cover of hairy grama tended (*P* = 0.08) to be greatest in spring-burned pastures, intermediate in summer-burned pastures, and least in autumn-burned pastures. Overall, these data demonstrated that prescribed fire timing was associated with minor changes to basal cover of certain C4 graminoid plant species.

Basal cover of total, native, introduced, annual, perennial or leguminous forbs did not differ (*P *≥ 0.12; Table 4) among spring-, summer-, or autumn-burned pastures; however, basal cover of nectar-producing forbs was greater (*P* = 0.02) in the autumn prescribed-fire treatment compared with the spring and summer prescribed-fire treatments. Similar trends have been observed in previous reports. Weir and Scasta (2017) reported that fires applied in September-October increased forb cover compared with fire applied at other times during the year, whereas Alexander et al. (2021) indicated that forb diversity was greater in plots burned in August or September compared with plots burned in April. Duncan et al. (2021) noted also that annual forbs and nectar-producing forbs were present in greater proportions in autumn-burned pastures compared with those burned in spring or summer. The consistency of these reports may indicate a potential habitat benefit of late summer or autumn prescribed burning to grassland-obligate invertebrates and the native birds that feed upon them (Ogden et al., 2019).

**Table 4.** Effects of prescribed-fire season on forb and shrub composition in native tallgrass prairie measured annually in June

	Prescribed fire season^1^				
Item, % basal plant cover	Spring	Summer	Autumn	SEM^2^	*P*-value^3^
Total forb cover	10.11	9.55	12.59	2.526	0.47
Native forbs	9.97	9.47	12.58	2.401	0.40
Introduced forbs	0.14	0.07	0.01	0.131	0.58
Annual forbs	0.39	0.56	0.98	0.258	0.12
Perennial forbs	9.77	9.01	11.66	2.386	0.53
Leguminous forbs	1.37	0.29	0.68	1.047	0.57
Nectar-producing forbs	1.46^b^	1.64^b^	2.66^a^	0.414	0.02
Sericea lespedeza	0.14	0.00	0.00	0.129	0.43
Total shrub cover	0.50^z^	1.20^y^	1.58^y^	0.438	0.06
Leadplant	0.48^z^	0.87^yz^	1.33^y^	0.376	0.10
New Jersey tea	0.01	0.21	0.01	0.112	0.14
Increaser shrubs^4^	0.02^z^	0.12^yz^	0.24^y^	0.092	0.08
Nectar-producing shrubs	0.48	1.09	1.34	0.400	0.11

^1^Eighteen pastures were grouped by watershed and randomly assigned to 1 of 3 prescribed-fire treatments: spring (11 April ± 5.7 d), summer (25 August ± 6.2 d), or autumn (2 October ± 9.0 d). Yearling beef cattle were grazed on all pastures from May to August at a targeted stocking density of 280 kg live-weight ha^−1^ following prescribed fire application.

^2^Mixed-model standard error of the mean (SEM) associated with comparison of treatment main-effect means.

^3^Treatment main effect.

^4^Shrubs that tend to proliferate in response to grazing (Vesk and Westoby, 2001).

^a,b^Within rows, means with unlike superscripts differ (*P* ≤ 0.05).

^y,z^Within rows, means with unlike superscripts tended to differ (*P* ≤ 0.10).

In our final analysis, sericea lespedeza basal cover was not different (*P* = 0.43; Table 4) between prescribed fire treatments. It was present in small amounts on all pastures before fire treatments were applied in 2018; however, it decreased to levels below detection by our third year of data collection on pastures burned in summer or autumn. Sericea lespedeza remained through the end of the experiment on pastures burned exclusively in the spring.

Basal cover of total shrubs tended (*P* = 0.06; Table 4) to be greater in summer- and autumn-burned pastures compared with spring-burned pastures. The trend for increased basal cover of shrubs in the autumn and summer prescribed-fire treatments was associated with changes in basal cover of leadplant and New Jersey tea. At the end of our experiment, basal cover of leadplant tended (*P* = 0.10) to be greatest in the autumn-fire treatment, intermediate in the summer-fire treatment, and least in the spring-fire treatment. In addition, basal cover of New Jersey tea was numerically greater (*P* = 0.14) in summer-burned pastures compared with spring- or autumn-burned pastures. The slight increase in basal cover of leadplant and new jersey tea, both desirable shrubs for livestock forage and wildlife habitat, contributed a minimum of 85% to total basal cover of all shrubs. Basal cover of increaser shrubs (i.e., shrubs that tend to proliferate in response to grazing) tended (*P* = 0.08) to be greater in autumn-burned pastures compared with spring-pastures; however, basal cover of increaser shrubs was small and accounted for less than 0.25% of total basal cover in autumn-burned pastures.

### Forage biomass accumulation.

Towne and Owensby (1984) reported that prescribed-fire timing influenced forage biomass production in the Kansas Flint Hills. Between 1968 and 1982, average forage biomass measured in October was greater in plots burned in mid-spring (10 April) or late spring (1 May) compared with plots burned in early spring (20 March) or winter (1 December). In our experiment, forage biomass accumulation measured in late June to early July did not differ (*P* = 0.58; Table 1) among prescribed-fire treatments. Similarly, Towne and Craine (2014) reported no differences in grass production at the end of the growing season among pastures burned in February, April, or November over a 20-yr period. In addition, July standing forage biomass did not differ between plots burned in April, August, or September (Alexander et al., 2021) or in pastures burned in April, August, or October (Duncan et al., 2021). We interpreted these data to suggest that prescribed fire applied at different time points throughout the growing season had minimal impact on forage biomass accumulation in the Kansas Flint Hills.

### Root carbohydrate reserves.

According to the report by Sowers et al. (2019), big bluestem, little bluestem, Indiangrass, and purple prairie clover made up a large portion (i.e., 46% to 69%) of the diets of grazing yearling cattle in the Flint Hills. These plant species represented a significant amount of pre-treatment plant cover at our study site (Duncan et al., 2021). Following prescribed-fire application, root starch and root water-soluble carbohydrate concentrations of big bluestem, little bluestem, Indiangrass, and purple prairie clover did not differ (*P* ≥ 0.26; Tables 5 and 6) among pastures burned in April, August, or October. Owensby et al., (1970) observed a rapid decline in available carbohydrates in rhizomes and stem bases of big bluestem from April to mid-May; however, when plant growth slowed, carbohydrate concentrations began to increase and peaked for non-grazed, burned plots in mid-June. We interpreted similar root starch and root water-soluble concentrations measured in mid-June to suggest that prescribed-fire timing in our experiment had minimal impacts on the ability of big bluestem, little bluestem, Indiangrass, or purple prairie clover to resynthesize root carbohydrates following periods of rapid growth.

**Table 5.** Effects prescribed-fire season on root starch concentrations (% DM) in big bluestem (*Andropogon gerardii*), little bluestem (*Schizachyrium scoparium*), Indiangrass (*Sorghastrum nutans*), and purple prairie clover (*Dalea purpurea*) during mid-June from 2018 to 2021

	Prescribed fire season^1^				
Item	Spring	Summer	Autumn	SEM^2^	*P*-value^3^
Big bluestem	2.25	2.78	1.89	0.685	0.44
Little bluestem	1.44	1.52	1.19	0.427	0.73
Indiangrass	2.65	1.83	1.64	0.920	0.51
Purple prairie clover	4.39	3.45	3.50	0.899	0.50

^1^Eighteen pastures were grouped by watershed and randomly assigned to 1 of 3 prescribed-fire treatments: spring (11 April ± 5.7 d), summer (25 August ± 6.2 d), or autumn (2 October ± 9.0 d). Yearling beef cattle were grazed on all pastures from May to August at a targeted stocking density of 280 kg live-weight ha^−1^ following prescribed fire application.

^2^Mixed-model standard error of the mean (SEM) associated with comparison of treatment main-effect means.

^3^Treatment main effect.

**Table 6.** Effects prescribed-fire season on root water-soluble carbohydratde concentrations (% DM) in big bluestem (*Andropogon gerardii*), little bluestem (*Schizachyrium scoparium*), Indiangrass (*Sorghastrum nutans*), and purple prairie clover (*Dalea purpurea*) during mid-June from 2018 to 2021

	Prescribed fire season^1^				
Item	Spring	Summer	Autumn	SEM^2^	*P*-value^3^
Big bluestem	3.33	4.29	3.93	0.585	0.26
Little bluestem	3.16	4.11	3.14	0.728	0.33
Indiangrass	4.83	3.59	4.02	0.991	0.45
Purple prairie clover	4.37	3.61	4.91	0.809	0.30

^1^Eighteen pastures were grouped by watershed and randomly assigned to 1 of 3 prescribed-fire treatments: spring (11 April ± 5.7 d), summer (25 August ± 6.2 d), or autumn (2 October ± 9.0 d). Yearling beef cattle were grazed on all pastures from May to August at a targeted stocking density of 280 kg live-weight ha^−1^ following prescribed fire application.

^2^Mixed-model standard error of the mean (SEM) associated with comparison of treatment main-effect means.

^3^Treatment main effect.

### Conclusions.

Shifting prescribed-fire timing from April to August or October reduced yearling stocker cattle weight gains by 5.1 to 6.3 kg over a 90-d grazing period and was associated with small but benign changes in rangeland plant composition. Conversely, prescribed fire timing did not influence forage biomass accumulation or root carbohydrate concentrations in key native tallgrass plant species. Overall, Flint Hills ranchers are encouraged to consider the costs associated with sericea lespedeza and yellow bluestem control using herbicides, as opposed to summer or autumn prescribed burning, versus the income sacrifice associated with small reductions in stocker-cattle growth performance.

## Supplementary Material

txad129_suppl_Supplementary_Material_1

txad129_suppl_Supplementary_Material_2
