# Supplementary material for: A 6-yr evaluation of prescribed-fire timing on yearling cattle growth performance and plant community dynamics on native tallgrass prairie in the Kansas Flint Hills
Source: Transl Anim Sci. 2023 Nov 18;7(1):txad129. doi: 10.1093/tas/txad129 (PMC10699836; doi:10.1093/tas/txad129)
Supplement: txad129_suppl_Supplementary_Material_2 [file txad129_suppl_supplementary_material_2.docx]

November 10, 2023

Dr. James Oltjen

Editor-in-Chief, Translational Animal Science

University of California, Davis, CA

Dr. Oltjen,

Please find attached the revised (R1) version of our manuscript entitled, “A six-year evaluation of prescribed-fire timing on yearling cattle growth performance and plant community dynamics on native tallgrass prairie in the Kansas Flint Hills”. We deeply appreciate your work and that of the reviewers of this manuscript. We’ve attempted to comply with all recommendations.

Thank you again for the opportunity to publish in Translational Animal Science. We look forward to receiving your comments.

Sincerely,


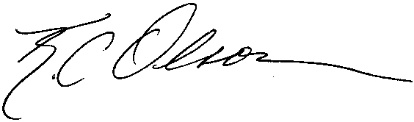


KC Olson

W.M. & F.A. Lewis Distinguished Professor

Animal Sciences & Industry

Kansas State University
