# Supplementary material for: A 6-yr evaluation of prescribed-fire timing on yearling cattle growth performance and plant community dynamics on native tallgrass prairie in the Kansas Flint Hills
Source: Transl Anim Sci. 2023 Nov 18;7(1):txad129. doi: 10.1093/tas/txad129 (PMC10699836; doi:10.1093/tas/txad129)
Supplement: txad129_suppl_Supplementary_Material_1 [file txad129_suppl_supplementary_material_1.doc]

Please note that all changes to the manuscript have been highlighted in yellow and with strike-throughs where relevant. Reviewer comments are in red text and our responses in black text below.

Response to Reviewer’s Comments

Translational Animal Science Manuscript 2023-1523

**Section Editor**

**General Comments**

“Well done, minor things to fix, see below.”

We thank the section editor for his kind words. We found several additional errors in composition, style, and form that were corrected. These are also highlighted in yellow.

A small error was discovered in the statistical analysis of forb basal cover (Table 4). Correction of the error resulted in very small changes to LS means, SEM, and P-values, but did not change the interpretation of the data. We regret this mistake.

**Specific Comments**

None.

********************************************************************************

Response to Reviewer’s Comments

Translational Animal Science Manuscript 2023-1523

**Reviewer 1**

**General Comments**

“The experiment is well-designed and the manuscript is well-written. Excellent discussion and reference to previous literature throughout. While I do not have a strong opinion, I suspect many ecologists may object to the relatively brief (5-year) application of treatments. After the introduction, impact of treatments on sericea lespedeza sort of gets lost/deemphasized. A few minor suggestions are provided below.”

We hope that the reviewer enjoyed reading our manuscript and we thank him or her for suggestions for improvement. In an ideal scenario, we would have liked to continue these burn treatments for another cycle of six years; however, we lacked the funding to do so. We believe that duration and scale (e.g., replication within year) of our experiment compares favorably with relatively recent research out of KSU and OSU and is substantially longer than publications that were used to establish the conventional combination of spring fire and intensive early stocking during the 1970s and 1980s. As to the lack of results and discussion regarding treatment effects on sericea lespedeza, we’ve added discussion to clarify that sericea lespedeza was present in all pastures during our pre-treatment year in 2018. By the end of the experiment, it was only present at detectable levels in spring-burned pastures.

**Specific Comments**

263 "Cover of yellow bluestem..."

The word cover was substituted for the word frequency.

265-267 “Previous indicates yellow bluestem was not affected by treatment, but here, you describe a year x treatment interaction.”

Although yellow bluestem was present on all pastures at the initiation of our measurements of basal cover in 2018, no treatment x time interaction (P = 0.68) was detected for yellow bluestem basal cover in our final analysis (2023).

271-282 “According to the introduction, sericea lespedeza was a major target for potential impact of prescribed fire timing treatments. Apparently, sericea was either not present in these pastures? Or it was not affected (as a leguminous forb). Suggest expanding the discussion to clarify if it was present and if so, why it was not affected by later season burns compared to other published literature.”

As with yellow bluestem, sericea lespedeza was present in small amounts on all pastures at the beginning of the experiment; however, it decreased to undetectable levels in summer- and fall-fire treatments after year 3 of our data collection but was still present on the spring-fire treatment through year 6. No treatment x time interaction was detected (P = 0.33) for basal cover of sericea lespedeza; therefore, the main effect of treatment was reported in Table 4. Some discussion was added (lines 286-290) to clarify this point.

276-277 “Seems like two sentences with "Similarly, ..." fits here better than "whereas".”

We ended up sticking with “whereas” in this case because there are several similar comparisons in series: 1) similar, 2) whereas, and 3) also. The thesaurus didn’t have a reasonable alternative.

Tables 5 and 6 “Somewhere in these tables, indicate sample timing.”

A description of sample timing was added to the titles of both tables.

********************************************************************************

Response to Reviewer’s Comments

Translational Animal Science Manuscript 2023-1523

**Reviewer 2**

**General Comments**

“Overall, a well written study. A large part of the focus of the introduction discussed sericea lespedeza. However, none of the study measured or shown the impact of timing of fire on SL, which I expected after reading the introduction. Why wasn’t it part of the species measurements? I assume lack of SL in the study cite?”

Through an oversight of the corresponding author, sericea lespedeza was not included in the original draft of Table 4.

No treatment differences in basal cover of sericea lespedeza were detected because of the small presence of the plant; however, we started our experiment with sericea in all pastures. It only remained in pastures treated with spring fire at the end of the experiment.

Sericea lespedeza has been added to Table 4 and a little discussion has been added in lines 286 – 290 for clarification.

**Specific Comments**

L12: “Seems to be more of a 5-year study since no treatment were applied or data collected in the “4th year”.”

It is true that fire treatments were withheld during year 4 because of uncooperative weather; however, animal performance and plant composition were still measured that year and reported in our manuscript. In practice, it is normal for beef producers to forego fire occasionally when conditions are too wet, too dry, or too windy.

L20: “Why after the start of grazing? If started collecting composition data in 2018, would your first year of data for summer and autumn burns be before burning and after burning for the spring burn? If so, can you really compare that first year of data?”

All grazing took place after fires were applied. Our pre-treatment plant data was collected in the summer of 2018. Our first fires were in August and October of 2018 and in April of 2019. Animal performance data was first collected during the spring and summer of 2019, following application of all fire treatments. This cycle was repeated a total of 6 times from 2018 to 2023.

L23: “When was it measured? In June each year?”

Specified in line 20.

L24: “suggest changing to “Average daily gain (ADG) was”

Changed as directed.

L35: ““Small influences” may not be the best wording. 0.05 to 0.07 kg/d over 90 days does add up to a decent group increase for the number of calves in this study or for a yearling operation.”

The reviewer is correct. An average difference of 11.5 to 13.9 pounds in total BW gain is not trifling at today’s prices but it’s still less than about 1.5% of final BW. This phrasing was intended to apply to effects on plant and animal data. We’ve tried to clarify our interpretation in the relevant R&D passages.

L80-82: “Citation for this sentence?”

The citation appears at the end of line 83.

L115: “Were calves allocated to treatment by BW? When was this? The first weight or on-trial weight?”

Calves were stratified by weight prior to pasture assignment. This has been clarified in line 115.

L116: “How long was your grower diet fed each year?”

Depending on year, the calves were fed the grower diet for between 2 and 3 weeks. We’ve added a minimum length of time to this passage.

L127: “Why do soil cover and botanical composition after grazing started each year?”

It is convention to wait to conduct most range plant assessments until all dominant species have expressed themselves for the year. Each has a unique growth cycle. Extremely juvenile plants can be difficult and time-consuming to identify. Much of this uncertainty can be avoided by waiting until grasses have reached the 3-leaf stage, which occurs around mid-June.

L327: “Did you actually measure impact of sericea lespedeza?”

We did. See explanation under response to general comments.
